# Practical Application of Anatomy of the Oral Cavity in Forensic Facial Reconstruction

**DOI:** 10.1371/journal.pone.0162732

**Published:** 2016-09-09

**Authors:** Paulo Eduardo Miamoto Dias, Geraldo Elias Miranda, Thiago Leite Beaini, Rodolfo Francisco Haltenhoff Melani

**Affiliations:** Laboratory of Forensic Anthropology and Odontology (OFLAB), Department of Social Dentistry, Dental School, University of São Paulo, São Paulo, Brazil; Seoul National University College of Medicine, REPUBLIC OF KOREA

## Abstract

The oral cavity’s importance in defining the facial region makes it a primary feature for forensic facial reconstruction (FFR). The aim of this study is to construct a pattern of reference for dimensions and proportions of the lips and establish parameters that may help estimate the vermilion borders’ height dimensions and the mouth’s width. By means of cone beam computed tomography, divided into two samples: sample 1 (n = 322; 137 male, 185 female) verified the linear distances delimited by anatomical landmarks in soft tissue. The sample 2 (n = 108; 40 male, 68 female), verified the proportions among the height of the vermilion borders, width of the mouth, and linear distances between craniometric landmarks in hard tissues, both from a Brazilian database. The measurements were completed using OsiriX, and the results were analyzed by means of descriptive statistics at a level of significance of 5%. The height of the vermilion borders corresponded to approximately 26% of the width of the mouth. The width of the mouth increased over the course of time in men and remained stable in women. In men, a mean intercanine distance of 75% of the total mouth’s width was found; for women, it was 80%. The parameters of the relations between soft and hard tissues in the oral cavity region presented that the distance between landmarks ID-SM (Infradentale-Supramentale) corresponded to 55% of the height of the vermilion borders of the mouth for both sexes, while the distance between landmarks PM-SD (Philtrum medium-Supradentale) corresponded to 85% in men and 88% in women. Mean values of 97% of the width of the mouth in women and 93% in men were attributed to the distance between the mentonian foramina. It was not possible to estimate the height of the labial vermilion borders by the bone measurements, FIs-Fli (Foramen incisivus superius-inferius) and NS-GN (Nasospinale-Gnathion). Profound knowledge of the anatomy and morphology of the oral cavity may contribute to increasing the precision of FFRs and help with human identification.

## Introduction

The skull forms a rigid structure to which soft tissues are attached [1]. One of the most notable regions of the face, irrespective of whether the interlocutor is speaking, is the mouth [2]. This region is associated with functions such as vocal communication, modes of expression, and attracting visual attention [3].

Forensic facial reconstruction (FFR), or facial approximation, is a technique that attempts to reproduce facial characteristics by means of study and modeling materials on the cranium surface, with the purpose of increasing the chances of recognition and possible identification [4]. FFR may be contextualized as a practical application of anatomy and forensic anthropology.

The techniques for reconstitution of the mouth, lips, and connected structures are partly based on dental measurements [5–7], which can be difficult to apply due to the postmortem loss of teeth [8]. Therefore, we present a task of interest to FFR: estimating the characteristics of the mouth based on the hard tissues, without exclusive support of tooth measurements.

The mouth is a vital part of the reconstructed face, and its position sheds light on the correct proportions of the face [6]. Some researchers have noticed a bias in mouth reconstruction, as there are no objectively proven efficient techniques for facial reconstruction [5–6, 9]. Although there has been research in this region—within the scope of anthropometrics—that has contributed to understanding the morphology of the mouth among different populations, few of these studies have analyzed the relationship between hard and soft tissues, as proposed in this study.

The aim of this study is to construct a pattern of reference for the dimensions and proportions of the lips and to estimate the height and width of the mouth by means of hard-tissue measurements in adults. These results can be used to establish support for FFR applications. Therefore, the authors expect to enhance the quality of FFRs, specifically in the oral cavity region, and increase the possibility of recognition and possible identification.

## Materials and Methods

### Sample

This was a cross-sectional observational study in which 815 cone beam computed tomography (CBCT) scans were analyzed. All of the exams were performed in an i-CAT (*Imaging Sciences International*, Hatfield–USA) tomograph, in which the patient remains seated in an upright position. They were instructed to keep a neutral facial expression and keep their mouths closed, with their lips lightly touching and in habitual occlusion. In this appliance, head positioning is aided by a chin cup that prevents movements during the exam. The CBCT were divided into two samples:

- Sample 1: Formed by 322 patients, 137 males and 185 female, with the mandible and maxilla in occlusion, healthy maxillary canines or natural roots, mouth in lip contact, a neutral facial expression, at least two central incisors present, and bone losses of up to one-third of the root length; the patients also presented all identified craniofacial landmarks. The sample 1 verified the linear distances delimited by anatomical landmarks in soft tissue. The study included individuals 11 years and older, because among this age range, a large part of lip growth and development already has attained dimensions close to those of a young adult by this age [10]. Age groups were divided among 11–19 years, 20–29 years, 30–39 years, 40–49 years, 50–59 years, and 60 years and older.
- Sample 2: Formed by 108 patients, 40 males and 68 female, the inclusion criteria were the same as those of Sample 1, except with regard to age. The age was based on maturation of the lips and their bone support; therefore, individuals over 20 years of age were included, since this is the age at which maturation of the vertical size of the vermilion borders occurs [11]. The sample 2 verified the proportions among the height of the vermilion borders, width of the mouth, and linear distances between craniometrics hard landmarks. The age groups analyzed were 20–40 years, 41–55 years, 56 years or older.

Excluded from the both samples were individuals with maxillary, mandibular, or bimaxillary edentulism; pathological craniofacial changes; maxillary fractures and evidence of orthognathic surgical or post-traumatic treatment with metal plates and screws; and obvious changes in soft tissue due to excessive compression on the chin cup of the tomograph.

### Location and anatomical landmarks placement

**Fig 1:**
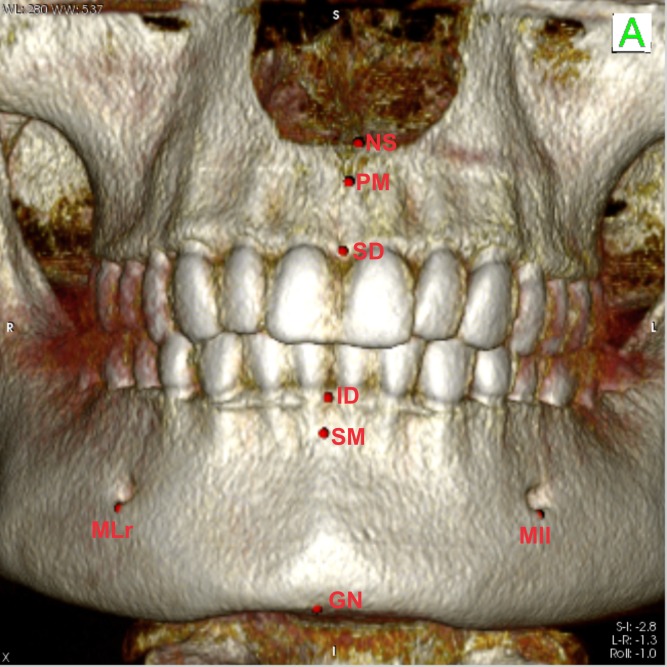
Location of skeletal landmarks NS, PM, SD, ID, SM, GN, MLr, and MLl, presented in OsiriX.

The Digital Imaging and Communications in Medicine (DICOM) files were imported into OsiriX software (Bernex, Switzerland), generating an initial previsualization of the scan by means of monoplanar reconstructions in the axial plane. It was possible to verify the extent of the CBCT and its conformity with the inclusion and exclusion criteria. In the 3D rendering mode, it was possible select the threshold for bone tissues to locate and place the craniometric landmarks [1, 12–13] of each sample (Table 1) by using the “point” tool (Fig 1).

**Table 1:** Anatomical landmarks in hard and soft tissues for linear measurements.

	Point	Abbreviation	Description
	Medians		
**1**	*Nasospinale*	NS	Lowest point on the inferior margin of the pyriform aperture, at the base of the nasal spine, projected in the sagittal plane
**2**	*Philtrum medium*	PM	In the maxilla, below the curvature of the anterior nasal spine
**3**	*Supradentale*	SD	Most anterior and superior point of the alveolar ridge, between the maxillary central incisors
**4**	*Infradentale*	ID	Most anterior and superior point of the alveolar ridge, between the mandibular central incisors
**5**	*Supramentale*	SM	On the midline, located in the depression between the mentonian eminence and the roots of the mandibular central incisors
**6**	*Gnathion*	GN	On the anterior border of the mandible that most projects downward in the sagittal plane
**7**	*Foramen incisivus inferius*	FIi	In the aperture of the incisive foramen, on the midpoint of the straight line delimited by the anteroposterior borders of this aperture, from the sagittal view
**8**	*Foramen incisivus superius*	FIs	In the aperture of the incisive canal in the nasal fossa, on the midpoint of the straight line delimited by the anteroposterior borders of this aperture, from the sagittal view
**9**	*Labiale superius*	ls	Midline point on the vermilion line of the upper lip
**10**	*Labiale inferius*	li	Midline point on the vermilion line of the lower lip
	Bilateral (r—right; l—left)		
**11**	*Canini*	CNr, CNl	On the vestibulodistal surface of the maxillary canines, at the interproximal contact height
**12**	*Mentale*	MLr, MLl	Most inferior point of the mentonian foramen
**13**	*Cheilion*	chr, chl	Located at the labial commissures

**Fig 2:**
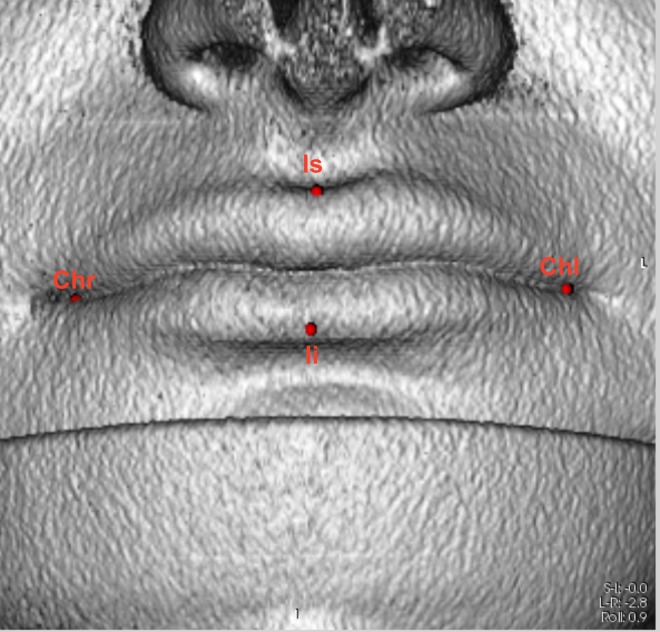
Location of facial landmarks chr, chl, ls, and li in the 3D rendering window presented in OsiriX.

Afterward, the surface threshold was changed in which objects with a radiodensity compatible with that of the skin were shown. In this mode, four of the anthropometric landmarks of the mouth were marked and located in accordance with the orientations for 3D volumes [1, 13] (Fig 2).

### Measurement of the linear distances and calculation of its proportions

**Fig 3:**
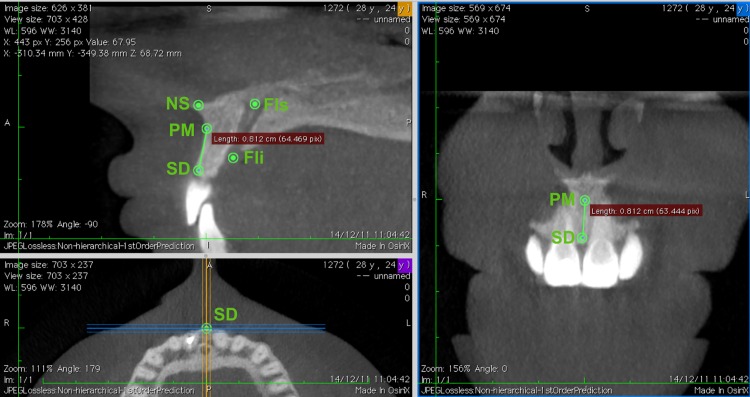
Example of coronal and sagittal planes that provide the same values for the same measures, in this case, PM-SD

For each measurement, in the 3D multiplanar reconstruction (3D MPR) mode, at the sagittal plane window, a straight line between two landmarks of interest was aligned precisely with the guide-line corresponding to the coronal plane (blue). After this, in the window of the coronal plane a real distance could be measured. The same straight line was aligned with the guideline in the sagittal plane (yellow). This was made to ensure the conformity of the measurements as different planes provided the same values for the same measures(Fig 3).

**Fig 4:**
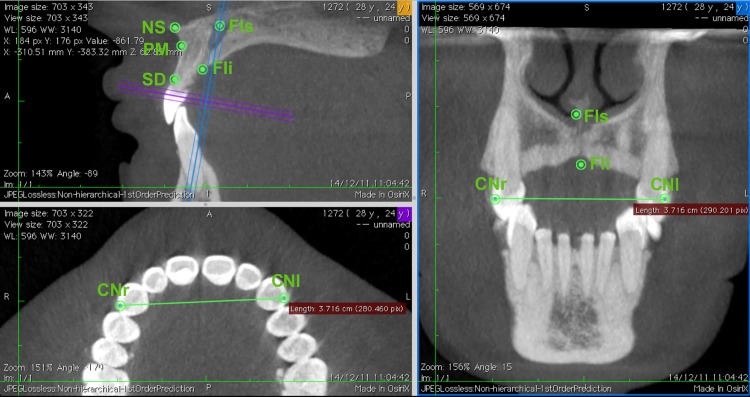
Example of axial (below to the left) and coronal planes (to the right) that provide the same values for the same measures, in this case, CNr-CNl

Thereby, the median landmarks were adequately aligned and could be measured in the window of the coronal plane. For the bilateral landmarks, the same process was performed in the window of the axial plane, with the measurement made in the window of the coronal plane (Fig 4).

Once the linear measurements were made with the “length” tool, these results were used to calculate the proportions between distances for the two samples (Table 2). The variables for the height of the vermilion borders and width of the mouth were compared between the sexes in both samples.

**Table 2:** Linear distances in mm and proportions in %, for hard and soft tissues.

	Measurement	Abbreviation	Description
	Sample 1		
**1**	*Labiale superius-inferius*	ls-li	Total height of the vermilion border of the lip
**2**	*Cheilion* right-left	chr-chl	Width of the mouth
**3**	Proportion ls-li/chr-chl	ls-li/chr-chl	Proportion between height of the vermilion border and width of the mouth
**4**	Proportion CNr-CNl/chr-chl	CNr-CNl/chr-chl	Proportion between the intercanine distance and width of the mouth
**5**	Expected value of chr-chl	(CNr-CNl x 100)/75	Expected value of the width of the mouth if the intercanine distance corresponded to 75% of its total value
	Sample 2		
**1**	*Foramen incisivus superius-inferius*	FIs-FIi	Length of the maxillary incisive canal trajectory
**2**	*Philtrum medium-Supradentale*	PM-SD	Height of the superior alveolar process
**3**	*Infradentale-Supramentale*	ID-SM	Height of the inferior alveolar process
**4**	*Nasospinale-Gnathion*	NS-GN	Height of the inferior third of the face
**5**	*Canini* right-left	CNr-CNl	Intercanine distance
**6**	*Mentale* right-left	MLr-MLl	Distance between the mentonian foramina
**7**	Proportion FIs-FIi/ls-li	FIs-FIi/ls-li	Proportion between the length of the incisive canal trajectory and the height of the vermilion borders
**8**	Proportion ID-SM/ls-li	ID-SM/ls-li	Proportion between the height of the inferior alveolar process and the height of the vermilion borders
**9**	Proportion PM-SD/ls-li	PM-SD/ls-li	Proportion of the height of the superior alveolar process and the height of the vermilion borders
**10**	Proportion NS-GN/ls-li	NS-GN/ls-li	Proportion between the height of the inferior third of the face and the height of the vermilion borders
**11**	Proportion MLr-MLl/chr-chl	MLr-MLl/chr-chl	Proportion between the distance between the mentonian foramina and the width of the mouth

In Sample 1, to evaluate whether the intercanine distance corresponded to 75% of the width of the mouth in mm [5], the authors included the variable “expected value of chr-chl–(CNr-CNl x 100)/75,” for comparison with the variable “width of the mouth–chr-chl.” This variable was calculated by the rule of three, attributing the percentage of 75% of its total value to the intercanine distance.

### Statistical Analysis

The statistical analyses were performed using the BioEstat program (Tefé, Brazil). A 5% level of significance was adopted, considering errors accepted of up to ± 1.0 mm for each measurement. The means and standard deviation for all of the linear measurements were obtained using descriptive statistics. The normality of the measurement values’ distributions was verified by the Kolmogorov-Smirnov test.

In the continuous quantitative variables with normal distribution, we verified the comparison of the means for the entire sample of the variables ls-li and chr-chl versus all of the age groups using the parametric t-test, Student’s t-test, and analysis of variance (ANOVA). For those samples that did not present normal distribution, the behavior was verified by the nonparametric Kruskal-Wallis test when three or more age groups were compared.

Intra- and interobserver calibration was performed using the reproducibility of the placement of the anatomical landmarks and variations in the measurements made. The results showed a less than 0.5 mm difference between landmarks and a 1.5 mm difference between the distances, implying excellent reproducibility [14] and results similar to another study that used the same OsiriX software [15].

### Ethical aspects

The CBCT images were provided by a radiology center and taken for the purpose of diagnosis and/or elective therapy. The images included information about the sex and age of the examined subjects. Written informed consent was not obtained; thus, patient information was anonymized and de-identified prior to analysis. This study was approved by the Research Ethics Committee of the Dental School, University of São Paulo, Protocol No. 129/11.

## Results

Sample 1 concluded that the height of the vermilion borders (ls-li) showed normal distribution and diminished with age for both sexes (p < 0.0001). The width of men’s mouths showed a tendency to increase over time. There was similarity between the means of the 11–19 and 20–29 age groups, as well as between the 20–29 and 30–39 age groups; however, there were statistically significant differences between those aged 11–19 and 30–39 (p < 0.01) and 20–29 and 40–49 (p < 0.05). For women, chr-chl had no normal distribution and demonstrated stability over time. There was a difference between the sexes (p < 0.01), with the men presenting mouths around 5 mm wider than those of women.

The proportion between the height of the vermilion borders and width of the mouth (ls-li/chr-chl) demonstrated a tendency to diminish with age for both men and women, accompanying the trend toward a reduction in the values of ls-li for both sexes and an increase in chr-chl for men. The mean values and standard deviation obtained for Sample 1, for men and women, are shown in Tables 3 and 4, respectively.

**Table 3:** Mean age in years, linear measurements in mm, and proportions in percentages for height of vermilion borders and width of the mouth, expected value for width in mm [5]; intercanine distance in mm; proportion between intercanine distance and width of the mouth in percentages and divided into age groups for men. SD = standard deviation.

Measurement		Age group (years)						
		11 to 19	20 to 29	30 to 39	40 to 49	50 to 59	60 to 76	Total
n		10	12	17	38	35	25	137
Mean age	Mean	15.10	23.75	34.12	45.34	54.06	65.40	45.74
	SD	2.08	3.05	2.37	3.06	2.72	4.58	15.08
ls-li	Mean	16.86	15.21	13.72	12.22	11.53	11.20	12.65
	SD	4.46	3.86	4.01	3.55	3.04	2.66	3.77
chr-chl	Mean	45.18	46.51	48.04	51.44	51.56	51.32	50.14
	SD	4.17	4.79	5.28	5.35	5.71	4.59	3.77
ls-li/chr-chl	Mean	37.50	36.25	30.24	24.34	22.60	22.04	26.20
	SD	9.68	10.03	7.60	6.57	6.70	5.73	8.81
(CNr-CNl x 100)/75	Mean	50.08	51.52	50.91	50.81	49.50	50.86	50.51
	SD	2.06	3.62	4.42	5.20	3.31	2.96	3.96
CNr-CNl	Mean	37.56	38.64	38.18	38.11	37.13	38.15	37.88
	SD	1.55	2.72	3.31	3.90	2.48	2.22	2.97
CNr-CNl/chr-chl	Mean	83.50	83.58	79.94	75.05	72.69	74.84	76.00
	SD	6.20	7.12	8.04	13.46	7.56	7.53	10.11

**Table 4:** Mean age in years, linear measurements in mm, and proportions in percentages for height of vermilion borders and width of the mouth, expected value for width in mm [5]; intercanine distance in mm; proportion between intercanine distance and width of the mouth in percentages and divided into age groups for women. SD = standard deviation.

Measurement			Age Group (years)						
			11 to 19	20 to 29	30 to 39	40 to 49	50 to 59	60 to 81	Total
	n		16	20	37	38	48	26	185
Mean age	Mean		14.88	24.80	35.00	45.00	53.67	65.23	43.30
	SD		2.66	2.78	2.45	3.22	3.09	5.26	15.12
ls-li	Mean		15.24	14.17	12.87	11.45	10.96	11.01	12.17
	SD		2.96	2.52	3.10	2.99	2.67	1.87	3.06
chr-chl	Mean		45.39	45.13	44.52	46.90	45.95	46.57	45.81
	SD		4.14	3.49	4.85	3.99	4.85	4.44	4.46
ls-li/chr-chl	Mean		33.75	31.60	29.27	24.63	24.15	23.69	26.80
	SD		6.72	5.83	7.87	7.29	5.85	3.89	7.27
(CNr-CNlx100)/75	Mean SD		49.652.85	34.9710.55	39.1411.37	45.387.90	47.763.54	47.963.47	44.338.80
CNr-CNl	Mean		37.24	36.66	36.64	36.13	35.64	35.97	36.24
	SD		2.14	1.64	2.18	2.61	2.21	2.60	2.31
CNr-CNl/chr-chl	Mean SD		82.567.89	81.505.17	83.2410.00	77.588.11	78.389.03	77.817.90	80.008.68

For men, there were no significant differences between the expected values for width of the mouth (mean of 50.51 mm) compared to the real mean (mean of 50.14 mm; p > 0.05). The difference between the means of chr-chl and (CNr-CNl x 100)/75 for the entire group was approximately 0.37 mm. Following this trend, in the proportion CNr-CNl/chr-chl, the authors noted that for men, the mean for the entire group was 76%.

For the female population there was a significant difference of approximately 1.5 mm between the means of the expected value and the value found. Then, for women, the intercanine distance represented 80% of the width of the mouth.

The results from Sample 2 for men and women are presented in Tables 5 and 6, respectively. In men, ls-li showed no normal distribution and did not vary significantly among the three age groups (p > 0.05). For women, the distribution also was not normal, but the oldest age group showed significant reduction in length (p < 0.01). The length of the trajectory between the incisive foramen and its aperture in the nasal fossa (FIs-FIi) had a proportion equivalent to 1.04 times the length of ls-li in males and 1.08 times in females.

**Table 5:** Mean age in years and linear measurements in soft and hard tissues in mm divided by age group for men.

Measurement		Age Group (years)			
		20 to 40	41 to 55	55 to 76	Total
	n	10	21	9	40
Mean age	Mean	30.50	49.43	65.44	48.30
	SD	6.13	4.95	7.06	13.46
ls-li	Mean	15.42	13.07	13.27	13.70
	SD	3.79	4.30	3.15	3.99
FIs-FIi	Mean	13.59	13.68	11.86	13.25
	SD	2.79	2.59	2.19	2.61
PM-SD	Mean	11.78	10.90	10.90	11.12
	SD	2.68	2.68	1.86	2.49
ID-SM	Mean	7.33	7.24	6.73	7.15
	SD	1.38	1.95	0.92	1.62
NS-GN	Mean	70.06	69.12	69.19	69.37
	SD	5.31	5.08	6.07	5.24
chr-chl	Mean	46.66	52.62	49.99	50.54
	SD	5.15	5.18	6.07	5.81
MLr-MLl	Mean	47.01	46.27	47.67	46.77
	SD	4.11	2.71	2.66	3.07

**Table 6:** Mean age in years and linear measurements in soft and hard tissues in mm divided by age group for women.

Measurement		Age Group			
		20 to 40	41 to 55	55 to 76	Total
	n	28	26	14	68
Mean age	Mean	31.64	49.77	64.00	45.24
	SD	6.09	3.99	6.70	13.73
ls-li	Mean	13.62	11.87	9.93	12.19
	SD	3.12	3.59	1.32	3.32
FIs-FIi	Mean	11.70	11.28	11.07	11.41
	SD	2.32	1.95	1.86	2.08
PM-SD	Mean	10.34	10.41	9.88	10.27
	SD	2.67	2.40	2.15	2.44
ID-SM	Mean	6.36	6.25	6.17	6.28
	SD	1.18	1.41	1.10	1.24
NS-GN	Mean	62.23	63.66	62.00	62.73
	SD	6.43	5.58	5.64	5.92
chr-chl	Mean	45.54	45.92	46.01	45.78
	SD	3.65	3.16	3.67	3.43
MLr-MLl	Mean	45.06	44.01	43.90	44.42
	SD	2.04	2.49	2.57	2.36

For the variable PM-SD compared to ls-li, the authors detected that for men, PM-SD assumed a value of approximately 85% of the height of the vermilion borders (Table 7). Comparing the value of PM-SD to ls-li multiplied by 0.85 demonstrated no differences between these means (p > 0.05; Table 7). For women, this proportion came close to 88% (p > 0.05; Table 8).

**Table 7:** Proportions between measurements in hard and soft tissues (in percentages) with means and SD. Calculation used for verifying them compared to the height of the vermilion borders and width of the mouth, p-value of the t-test, and interval of confidence (IC95%) of the verification in mm for men.

Men	n = 40			
Measurement	Mean	Verification	t-Test	IC
	SD			
FIs-FIi/ls-li	104.96	ls-li x 1.04 compared to FIs-FIi	p> 0.05	-0.59 to 2.60
	37.08			
ID-SM/ls-li	55.45	ls-li x 0.55 compared to ID-SM	p> 0.05	-1.09 to .31
	18.07			
PM-SD/ls-li	85.91	ls-li x 0.85 compared to PM-SD	p> 0.05	-0.54 to 1.60
	25.89			
NS-GN/ls-li	544.07	ls-li x 5.4 compared to NS-GN	p> 0.05	-2.06 to 11.31
	142.54			
MLr-MLl/chr-chl	93.68	chr-chl x 0.93 compared to MLr-MLl	p> 0.05	-1.62 to 2.07
	11.73			

**Table 8:** Proportions between measurements in hard and soft tissues (in percentages) with means and SD. Calculation used for verifying them compared to the height of the vermilion borders and width of the mouth, p-value of the t-test, and IC95% of the verification in mm for women.

Women	n = 68			
Measurement	Mean	Verification	t-Test	IC
	SD			
FIs-FIi/ls-li	108.46	ls-li x 1.08 compared to FIs-FIi	p< 0.05	-2.60 to 0.89
	28.70			
ID-SM/ls-li	55.00	ls-li x 0.55 compared to ID-SM	p> 0.05	-0.07 to 0.92
	16.54			
PM-SD/ls-li	88.35	ls-li x 0.88 compared to PM-SD	p> 0.05	-0.27 to 1.18
	25.39			
NS-GN/ls-li	546.62	ls-li x 5.4 compared to NS-GN	p> 0.05	-1.11 to 7.29
	134.21			
MLr-MLl/chr-chl	97.58	chr-chl x 0.97 compared to MLr-MLl	p> 0.05	-1.01 to 0.98
	9.08			

The ID-SM distance for both sexes was equivalent to approximately 55% of the total value of ls-li. Verification was completed by calculating 55% of ls-li and comparing it to the values of ID-SM, showing no significant differences (p > 0.05; Tables 7 and 8).

The mean height of the inferior third of the face compared to the height of the mouth’s vermilion border showed an approximate value of 5.4 times that of ls-li. When verifying, by multiplying the measurements of ls-li by 5.4 and comparing them to those of NS-GN by the t-test, the authors found no statistically significant discrepancy (Tables 7 and 8).

The ratio between MLr-MLl/chr-chl in men was approximately 93% (Table 7), whereas for women, the ratio was approximately 97%, without differences in verification (Table 8).

## Discussion

In this article, the authors studied the anatomy of the oral cavity and its relations with the bone structures for application in FFR. The results demonstrated that the height of the vermilion borders tended to diminish with age, in agreement with previous research [16]. An Italian sample [10] presented even higher values for lip height than those of Brazilians in the same age group. In a similar manner, a difference was noted for ls-li in Chinese and Korean subjects [17], who presented ls-li values approximately 2 mm larger than Brazilians in the age ranges between 11 and 29 years, with this discrepancy being closer to 3 mm between the ages of 30 and 40 years.

The reduction in ls-li may be physiologically attributed to a reduction in the mobility and elasticity of the lips, translated into an increase in the cutaneous portion of the upper lip and consequent reduction in the vermilion border; that does not imply a reduction in volume, in spite of the thickness diminishing as well [18]. Although there is some difference between the ages of the lower lip’s maturation, which continues to grow up until the age of 18 years in men [10], this difference may have diminished as a result of this growth, representing a few millimeters, and the height of the vermilion borders having been collected between ls and li, which may have diminished this discrepancy because the lips were not considered individually.

Although no differences in the ls-li values were detected between men and women, in agreement with other studies [19–20], statistical analysis pointed out different heteroscedastic variances. This does not imply that the ls-li values between men and women are statistically different, but this heteroscedasticity suggests that separate analysis would be more adequate to document the anthropometric data of each population. Other studies found differences between the genders, whether for ls-li or other variables, and in which the male values repeatedly assumed higher values [1,10,16–17,21].

In the male sample of this research, the width of the mouth presented a tendency to increase with the passage of time, in a similar manner to that of Caucasian Italians [16]. In this same group, the width of chr-chl in women also tended to diminish, whereas in the Brazilian sample, it remained stable. The Brazilian mean values for both sexes were discretely higher than those detected for young Chinese and Korean subjects [17] and similar to other studies [1, 5, 10, 14]. Similarly, men presented a higher mouth width value compared to women [19, 21], with a mean difference of up to 5 mm. Although some parameters showed similarity to those of foreign populations, others differed, even if discretely. In view of these differences and similarities, whether they were subtle or not, the authors noted that the anthropological characteristics of the Brazilian sample was different from the others documented by the literature. Therefore, further population studies, such as the present study, are important.

The collection of anthropological information for Brazilians becomes particularly complex when considering skin color. Skin color is variable and can be affected by conditions such as albinism. Even among individuals of the same anthropological type, such as Caucasians, differences may be detected in the soft tissues of the inferior third of the face in Brazilians when compared to North Americans [22]. Regarding the thickness of facial soft tissues, studies have shown that there were no differences between the Brazilian anthropological types [4]. Thus, this study, which intended to collect information that may help characterize FFRs performed on individuals in a complex, miscegenated population [23], is in agreement with other Brazilian studies [4, 24].

Although the mean ratio between ls-li/chr-chl of 26% was maintained, it showed a tendency to diminish over time, ranging between 38% and 22% in men and 34% and 24% in women. For the same age groups in Caucasian Italians [16], the variation was similar for men, 35% to 21%, and in women, there were ranges between 35% and 19%, indicating that some age ranges had lower mean values than those of Brazilians, especially those over 40 years of age. The age groups between 20 and 40 years also showed similar proportions to those of young Caucasian Italians [10, 25].

In spite of ls-li/chr-chl appearing to be a generalized parameter, it should be observed that they are means for a researched sample, and FFRs may start with these general principles and follow the trends within the standard deviation for each variable, such as, for example, the reduction in ls-li with age in both genders or the increase in chr-chl after the age of 40 years in men. The FFRs completed on the basis of this study will not necessarily have the proportion of 26% between ls-li and chr-chl, but rather will start from this point and may be individualized according to the tendency of the age group of the skull being reconstructed.

Regarding the actual and expected values of chr-chl [5], the authors found that the methodology that recommends the intercanine distance as equivalent to 75% of the width of the mouth had results applicable to Brazilian men, while in women, an adjustment to 80% demonstrated better results. In the reference study [5], the sample used was Asiatic men (n = 12) and women (n = 17); European men (n = 17) and women (n = 44); and persons of other origins (n = 5), totaling 93 individuals, not divided according to age group, considering the mean values. The same was done in this study but with a larger sample. There was no apparent reason why chr-chl did not change over time in females of this sample.

In FFR, the width of the mouth may be estimated by the interpupillary distance, the distance between the medial borders of the iris, or the intercanine distance itself [6]. An evaluation of these methods compared to the real measurement [26] demonstrated that although the second method may provide approximate results, the other two may provide under- or overestimated values. A more effective alternative attributed a value to the width of the mouth as the intercanine distance plus 57% of the distance between the distal portion of the canines and the center of the pupils [26].

The methods that detected relations between the width of the mouth and other soft tissues are valid, but they are extremely critical for application in FFR. The reconstruction of soft tissue that will serve as reference for estimates may be subject to errors that cumulatively may introduce imprecisions in the subsequent stages of reconstructing the width of the mouth. Questions about the effectiveness [27] of the methods for positioning the eyeballs [28, 29], for example, place the effects that these discrepancies may cause in estimating the width of the oral cavity in doubt, based on these parameters.

In FFR, the more the soft tissue characteristics can be estimated from the stable, measurable, hard tissues, the better. Thus, the choice of the canine teeth as the starting point for estimating the width of the mouth was considered a parameter. Adjustment of the technique [28] interpreted by Wilkinson [6] and proposed by Stephan [26] demonstrated results with good precision. However, the problem of postmortem tooth loss may make it unfeasible to apply the technique. In this study, the authors found that in the same way, antemortem tooth losses in the sample were less frequent in the canines than in other groups of teeth. In light of the canine teeth’s resistance to avulsion caused by putrefaction of the periodontal ligament, they may be considered adequate predictors of the width of the mouth in FFR.

Knowing the width of chr-chl—estimated by 75% and 80% of its value attributed to CNr-CNl for men and women, respectively—the height of the labial vermilion borders may be estimated as 26% of this width. In possession of this information, the expert can guide the FFR, knowing the approximate limits occupied by the mouth. In spite of the estimate of chr-chl alone not allowing the asymmetry of the mouth to be evaluated in relation to the midline, the position of the mouth reconstructed on this line may contribute to a reconstruction with a good chance of resemblance to the target individual, at least as far as the limits of size occupied are concerned.

In the same way, reconstruction of the mouth slit at an approximate height of the inferior incisive line may help to position the limits of the vermilion border height on its midline. The authors noted that in spite of ls-li being the most extensive point of the vermilion borders along the midline, laterally, they may be higher, even if subtly, due to the position of the base of the philtrum, delimited by the bilateral *christa philtri* landmarks [1]. Reconstruction of these points is difficult in FFR, because they have little relation to the adjacent hard and soft tissues, making it difficult to estimate the shape of the filter and add some degree of subjectivity to the method.

In Sample 2 the authors sought to investigate hard-tissue parameters that could help estimate the dimensions of the mouth in order to avoid or minimize problems related to the reconstruction of soft tissues that begin with the reconstruction of other soft tissues. Furthermore, the authors tried to choose bone measurements that could be more stable and less subject to alterations over the course of an individual’s life. No dental measurements were chosen due to possible problems linked with postmortem tooth loss.

The choice of the FIs-FIi measurement was based on these parameters. In addition, there are no studies to test the hypothesis of a relation between FIs-FIi and ls-li. Since the trajectory of the incisive canal accompanies downward growth of the maxilla [30], and ls-li tends to increase in size between childhood and adolescence [1, 10, 18], the authors opted to test the hypothesis that there was a relationship between FIs-FIi and ls-li in an experimental manner. In spite of the authors finding a mean size of FIs-FIi almost equal to that of ls-li (104% in men and 108% in women), verification of this datum pointed out a wide interval of confidence, greater than 2 mm (Tables 7 and 8) this parameter may be imprecise for estimating ls-li.

In the same way, the authors decided to test the proportions between the height of the inferior facial third bone (NS-GN) and the height of the vermilion borders (ls-li), seeing that the facial third presents an increase in the first decades of life and afterward diminishes [31], similarly to ls-li [1, 10, 16]. Furthermore, the landmarks NS and GN are easily located on the dry skull. The authors also compared this measurement in an experimental manner, because the articles studied the facial height proportions measured in soft tissues [20, 25]. There were no statistical differences between the values of NS-GN and ls-li multiplied by 5.4; however, the rather wide interval of confidence makes the precision of this measurement inefficient for estimating ls-li, contraindicating its use for this purpose (Tables 7 and 8).

For PM-SD and ID-SM, the authors attempted to verify proportions based on the growth of alveolar processes and their reduction with age [21,30] versus the similar tendency of the height of the vermilion borders, li [1, 10, 16]. The authors found a narrower interval of confidence that may make the estimate of ls-li applicable by attributing 85% of its value to PM-SD in men and 88% of its value to PM-SD in women. In the same manner, the value of ls-li may be estimated by attributing 55% of its value to ID-SM for both genders.

As these values were measured in individuals with at least one of the central incisors present and bone losses of up to one-third of the root length, the applicability of this finding for skulls outside of this profile must still be tested in other research. In cases of postmortem losses of the central incisor teeth, evaluation of the level of bone loss may become complicated, which requires caution from the expert. A positive aspect of the results for PM-SD and ID-SM is that should there be ante- or postmortem tooth losses in only one of the dental arches, the estimate may still be made by using the measurement of the opposite arch.

For the width of the mouth, chr-chl may be attributed to 93% of the value of MLr-MLl in men and 97% in women. With an interval of confidence discretely greater than 2 mm in men, caution is required in the application of this proportion, setting it aside if other parameters, such as the intercanine distance, can be used. On the other hand, the use of the mentonian foramina may be an alternative in skulls without maxillary canines with significant bone losses. The applicability of this parameter in edentulous individuals may also be considered as an alternative to estimating chr-chl by the reference in the infraorbital foramina [7].

## Conclusions

The height of the vermilion borders tended to diminish over time in both sexes, while the width of the mouth increased in men and remained stable in women. The height of the vermilion borders corresponded to 26% of the width of the mouth on average. The intercanine distance (CNr-CNl) corresponded to 75% of the width of the mouth in men and 80% in women.

The parameters of the relations between soft and hard tissues in the oral cavity region showed that the distance between landmarks ID-SM corresponded to 55% of the height of the vermilion borders of the mouth for both sexes, while the distance between landmarks PM-SD corresponded to 85% in men and 88% in women. The distance between the mentonian foramina corresponded to 97% of the width of the mouth in women, while in men, this value was 93%, with lower precision. It was not possible to estimate the height of the labial vermilion borders by the bone measurements FIs-Fli and NS-GN.

These anatomical and morphological findings of the oral cavity region may contribute to increasing the precision of FFRs and help with human identification.
